# Comprehensive miscarriage dataset for an early miscarriage prediction

**DOI:** 10.1016/j.dib.2018.05.012

**Published:** 2018-05-17

**Authors:** Hiba Asri, Hajar Mousannif, Hassan Al Moatassime

**Affiliations:** a OSER Laboratory, Cadi Ayyad University, Marrakech, Morocco; b LISI Laboratory, Cadi Ayyad University, Marrakech, Morocco

## Abstract

We present risk factors for predicting miscarriage. Our data is created through an android mobile application that collects automatically real-time data about the pregnant woman. This process is done every 60 s while the mobile application is on active mode. We distinguish two types of data: data from mobile phone and data from healthcare sensors. Data generated is real and concerns real pregnant women to test and validate the proposed system and assess its performance and effectiveness.

## Specifications Table

**Table:** 

Subject area	*Healthcare & Computer Science*
More specific subject area	*Predictive and preventive medicine*
Type of data	*Text file.*
How data was acquired	*Data is acquired from mobile phone and healthcare sensors.*
Data format	*Analyzed*
Experimental factors	
Experimental features	*Age, Heart Rate Variability, BMI, History of Previous Miscarriage, Activity, Location, Body Temperature, Body Mass Index (BMI), Stress motion, Blood Pressure, Weight, Height*
Data source location	*Marrakech, Morocco.*
Data accessibility	*The dataset is available on GitHub platform via the following link:*https://github.com/hibaasri/Miscarriage-Prediction

## Value of the data

• Data is of value to the researches because it is a real data generated.
• Data can be used in the development of other experiments in healthcare area.
• Data can be used for comparing efficiency and effectiveness of data mining algorithms in predicting outcomes.
• The volume of data is prominent for accurate results.
• Data can be used as a benchmark for other researchers for making real test and validate their results.

## 1 Data

The data includes all risk factors of miscarriage that the mobile application generates from healthcare sensors and mobile phone. The Dataset contains risk factors of miscarriage, patient's personal information and data's file: Age, Heart Rate Variability (BPM), History of Previous Miscarriage (nmisc), Activity, Location, Body Temperature (Temp), Body Mass Index (BMI), Stress motion (stress), Blood Pressure (BP), Weight, Height, Email address, File's Type, File's Saved Time and File's Identifier. All risk factors data are in numeric type for analytical reasons.

## 2 Experimental design, materials, and methods

Different sources are used to get the data: Mobile phone and healthcare sensors. Table 1 presents a description of each attribute of our dataset.

• Data from sensors:

– Heart rate variability [1],
– Stress and blood pressure [2],
– Temperature variation [3],
– Physical Activity [4].

• Data from mobile phone:

– BMI [5],
– Weight,
– Height,
– Number of previous miscarriages,
– Maternal age [6],
– Location [7],
– Actual activity [4].

**Table 1:** Miscarriage dataset attributes.

	Attribute	Type	Description
1	ID	Integer	The key of JSON document.
2	Activity	Integer	The level of the activity of the woman during the day.
3	Location	Integer	Location where the woman spends her time.
4	BMI	Double	Body Mass Index: It is an attempt to quantify the amount of tissue mass (muscle, fat, and bone) in an individual, and then categorize him/her.
5	nMisc	Integer	The number of previous miscarriages of the woman during her pregnancies.
6	Age	Double	The maternal age of the woman.
7	Weight	Double	The weight of the woman: The quantity of heaviness or mass. It is used in BMI calculation.
8	Height	Double	The height of the woman. It is used in BMI calculation.
9	Temp	Double	Body Temperature of the woman.
10	BPM	Long	Heart Rate Variability (HRV) per minute.
11	Stress	Long	Stress Emotions.
12	BP	Long	Blood Pressure indicator.
13	Time	String	The time to save the file in the database server.
14	User_email	String	The ID of the woman to whom belongs the current document. It is used to extract the right data about woman.
15	Type	String	The type of document. It is used to differentiate between authentication documents and documents that contain prediction attributes.

Attributes like Weight, Height, maternal age and number of previous miscarriage are collected via a registration form that the patient fills during his first use of mobile application. Location data is collected via GPS mobile tool [8], while actual activity is detected through a predefined machine learning library on android. The BMI is calculated based on height and weight values.

**Fig. 1:**
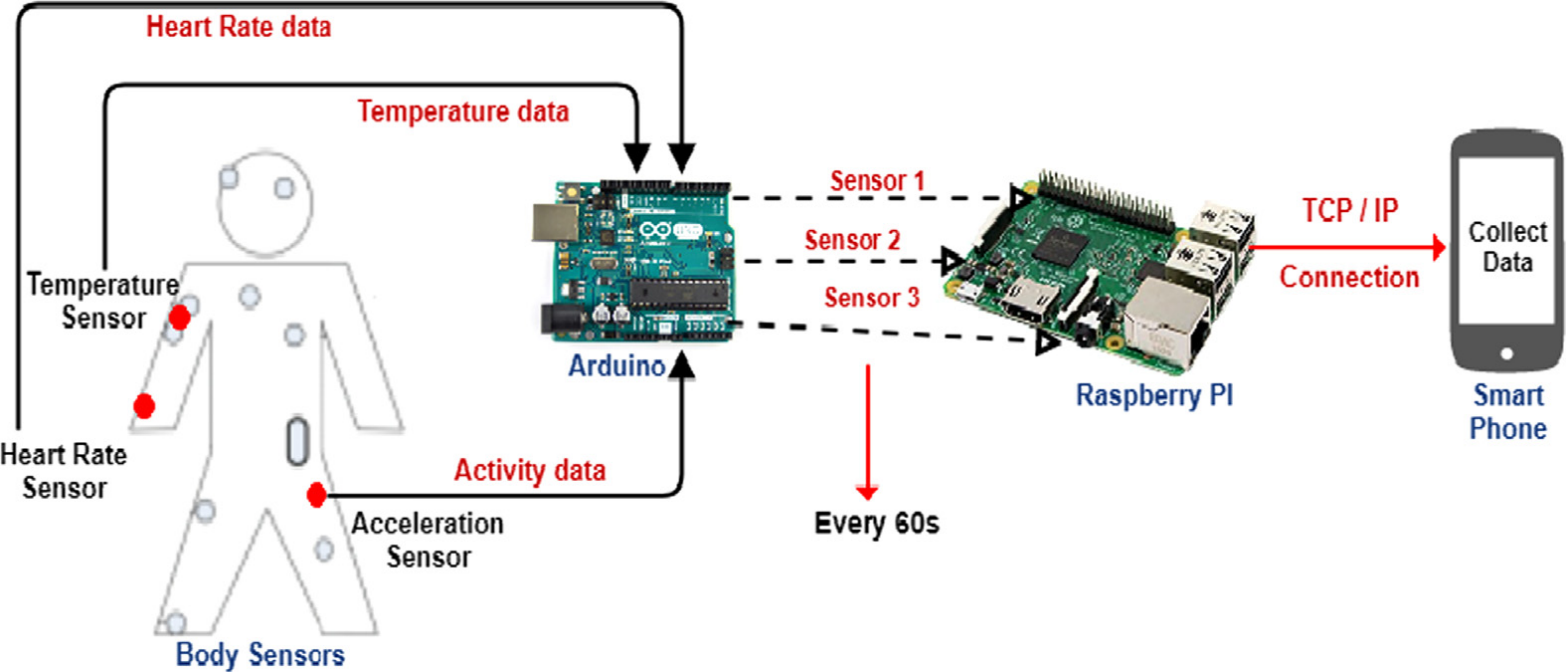
Gathering sensors data workflow.

Data from sensors are collected using a microprocessor ARDUINO UNO as it contains many input for linking wires of sensors and sent to Raspberry Pi 3 which is a Nano-computer where process is done [9] (see Fig. 1). It collects data every 60 s and send it to our mobile phone application to be analyzed in a Big Data Platform.
