# Supplementary material for: Comprehensive miscarriage dataset for an early miscarriage prediction
Source: Data Brief. 2018 May 17;19:240–3. doi: 10.1016/j.dib.2018.05.012 (PMC5992995; doi:10.1016/j.dib.2018.05.012)

Conflict of Interest Form

We wish to confirm that there are no known conflicts of interest associated with this publication and there has been no significant financial support for this work that could have influenced its outcome.

We confirm that the manuscript has been read and approved by all named authors and that there are no other persons who satisfied the criteria for authorship but are not listed. We further confirm that the order of authors listed in the manuscript has been approved by all of us.

Declarations of interest: none

Authors:

Hiba Asri

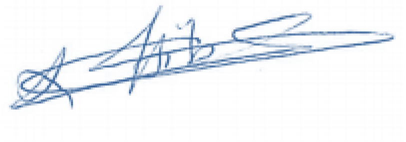

Hajar Mousannif

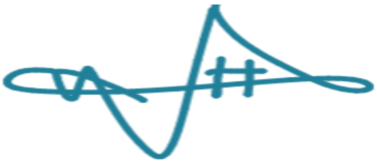

Hassan Al Moatassime

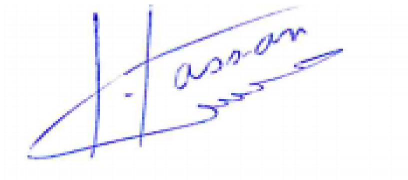

Supplement: Supplementary file 1 — Supplementary material [file mmc1.pdf]
